# Impact of Antiglaucoma Drug Number and Class on Corneal Epithelial Thickness Measured by OCT

**DOI:** 10.3390/ph18060868

**Published:** 2025-06-11

**Authors:** Piotr Miklaszewski, Anna Maria Gadamer, Dominika Janiszewska-Bil, Anita Lyssek-Boroń, Dariusz Dobrowolski, Edward Wylęgała, Beniamin Oskar Grabarek, Michael Janusz Koss, Katarzyna Krysik

**Affiliations:** 1 Department of Ophthalmology, St. Barbara Hospital, Trauma Centre, 41-200 Sosnowiec, Poland; annagadamer978@gmail.com (A.M.G.); dominika.bjaniszewska@gmail.com (D.J.-B.); anitaboron3@gmail.com (A.L.-B.); dardobmd@wp.pl (D.D.); kkrysik@gmail.com (K.K.); 2 Department of Ophthalmology, Faculty of Medicine, Academy of Silesia, 40-555 Katowice, Poland; 3 Collegium Medicum, WSB University, 41-300 Dabrowa Gornicza, Poland; bgrabarek7@gmail.com; 4 Department of Ophthalmology, District Railway Hospital, 40-760 Katowice, Poland; wylegala@gmail.com; 5 Department of Ophthalmology, Faculty of Medicine, Medical University of Silesia, 40-555 Katowice, Poland; 6 Augenzentrum Nymphenburger Höfe, 80335 Munich, Germany; koss@augenarzt-muc.de; 7 Department of Ophthalmology, Augenklinik der Universität Heidelberg, 69120 Heidelberg, Germany; 8 Augenklinik Herzog Carl Theodor, 80335 Munich, Germany

**Keywords:** corneal epithelial thickness, glaucoma, antiglaucoma therapy, optical coherence tomography, benzalkonium chloride, latanoprost, ocular surface disease, polypharmacy

## Abstract

**Background/Objectives:** The corneal epithelium plays a vital role in maintaining corneal transparency and ocular surface integrity. Chronic topical use of antiglaucoma medications may induce epithelial changes, especially with the concurrent use of multiple agents. This study aimed to evaluate the association between the number and class of antiglaucoma medications and central corneal epithelial thickness (CET), measured using a spectral-domain optical coherence tomography (SD-OCT) device. **Methods:** This cross-sectional study included 456 eyes from 242 adults (median age 72 years), grouped by the number of antiglaucoma agents used (0–4 medications). All pharmacologically treated participants had received the same regimen for ≥6 months. CET was measured using SD-OCT (SOLIX, Optovue). Generalized estimating equations (GEEs) accounted for inter-eye correlation. Two models were constructed: one evaluating specific medication effects and another assessing CET reduction per additional drug used. Age and sex were included as covariates. **Results:** CET progressively decreased with the number of medications, ranging from 53 µm in controls to 48 µm with quadruple therapy. Multivariable GEE analysis confirmed a cumulative thinning effect, with each additional medication associated with further CET reduction (β = −2.83 to −9.17 µm, *p* < 0.001). Latanoprost exerted the most pronounced single-drug effect (β = −3.01 µm, *p* < 0.001). Age was a modest negative predictor, while sex showed no significant effect. **Conclusions:** The cumulative number and specific class of antiglaucoma medications have a significant impact on corneal epithelial thickness. These results emphasize the need for vigilant ocular surface evaluation in patients on multi-drug regimens and propose CET as a surrogate marker for the burden of topical therapy.

## 1. Introduction

The cornea is a critical structure of the eye, essential for maintaining its optical properties and serving as a protective barrier [1,2]. Anatomically, it comprises five distinct layers: the epithelium, the Bowman’s layer, the corneal stroma, Descemet’s membrane, and the endothelium [3]. The epithelium consists of 5–6 layers and includes three types of cells: superficial cells, wing cells, and basal cells, each with specific roles in structure and function [4]. The corneal epithelium is a highly active, self-renewing layer, with epithelial cells having a lifespan of approximately 5 to 7 days [5]. The corneal epithelium, a stratified squamous epithelium, serves as the first line of defense against mechanical injury, pathogens, and toxins [6]. It maintains transparency and provides a smooth refractive surface crucial for optimal vision. Optical coherence tomography (OCT) has become an indispensable tool in ophthalmology, allowing for the precise assessment of corneal layers. Its non-invasive nature and high-resolution imaging enable the early detection of pathological changes in the epithelium, which was previously only possible with histopathological examination. This provides a critical advantage in managing patients with systemic and ocular diseases [7]. The study of corneal epithelial thickness provides valuable insights into corneal health and early pathological changes in its structure. This is particularly relevant in glaucoma, where the long-term use of medications may lead to epithelial damage [8,9]. In their study, Li and colleagues evaluated the corneal epithelial thickness in healthy individuals using Fourier-domain OCT (FD-OCT) and reported that the mean epithelial thickness in the central cornea was 52.3 ± 3.6 μm [9].

Glaucoma refers to a group of progressive optic neuropathies characterized by the degeneration of retinal ganglion cells and the retinal nerve fiber layer, resulting in changes in the optic nerve head [10].

Glaucoma represents the second leading cause of blindness globally [8]. The reduction in intraocular pressure (IOP) remains the only proven therapeutic approach for glaucoma management, with ocular hypotensive drops serving as the primary method for achieving pressure control [11]. Despite existing discrepancies in estimates of the number of people affected by glaucoma, it is generally accepted that the condition affects a small percentage of the population, depending on the ethnic group. In certain populations, particularly among older individuals, the prevalence can reach as high as 12%. Evidence indicates a steady increase in the number of individuals diagnosed with glaucoma, resulting in an expanding demand for therapeutic interventions and pharmacological management [12,13,14].

Glaucoma eye drops are associated with both local and systemic adverse effects. Local effects include, among others, punctate keratitis, corneal erosion, conjunctival allergy, conjunctival hyperemia, eyelash elongation, eyelid and iris pigmentation, eyelid retraction, contact dermatitis, hyperpigmentation, prostaglandin-associated periorbitopathy, eyelash depigmentation, and hypertrichosis (excessive hair growth on the skin). Benzalkonium chloride (BAK), commonly present in many formulations, has been shown to exacerbate ocular surface damage, leading to chronic conjunctivitis and dry eye syndrome through its toxic effects on the corneal and conjunctival epithelium. Systemic adverse effects, particularly those associated with β-blockers, include bradycardia, hypotension, arrhythmia, and asthma exacerbations [15,16,17].

The purpose of this study was to conduct a detailed analysis of corneal epithelial thickness in healthy individuals and glaucoma patients using OCT. Particular emphasis was placed on evaluating the impact of the number of antiglaucoma medications on changes in the epithelial thickness across different corneal zones. A key objective was to determine whether a correlation exists between the number of antiglaucoma medications used and changes in CET. Comparing the results between glaucoma patients and healthy subjects, our aim was to assess how the long-term use of IOP-lowering eye drops affects the structure of the corneal epithelium.

## 2. Results

### 2.1. Cohort Characteristics

The study cohort comprised 242 adult patients, with 456 eyes analyzed to evaluate the central corneal epithelial thickness (CET) among individuals using antiglaucoma eye drops compared with a control group of healthy individuals. At the patient level, the cohort was predominantly female (58.26%, *n* = 141) compared with male (41.74%, *n* = 101), with a median age of 72 years (IQR: 68–78 years). Age distributions varied by sex, with males having a median age of 70.5 years (IQR: 66–74 years) and females a median age of 75 years (IQR: 70–82 years). When stratified by the number of medications used, median ages were relatively consistent across groups: 72 years (IQR: 69.5–76.5) for the control group (0 medications), 71 years (IQR: 66–79.5) for monotherapy, 73 years (IQR: 69–78) for dual therapy, 73 years (IQR: 72–79.25) for triple therapy, and 71.5 years (IQR: 70–75.75) for quadruple therapy. At the eye level, the median CET across all 456 eyes was 51.0 µm (IQR: 48.0–54.0 µm). No missing data were observed for the reported variables, ensuring completeness of the dataset.

### 2.2. CET by Number of Medications

**Figure 1:**
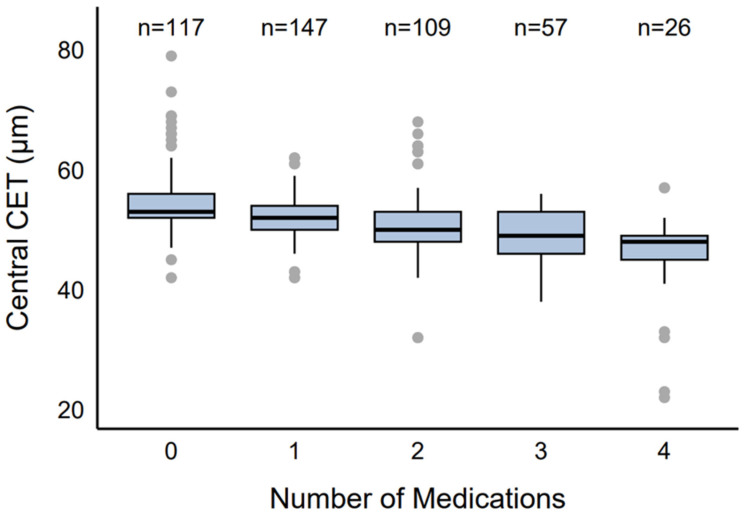
Distribution of CET by number of antiglaucoma agents.

CET varied by the number of medications used (Figure 1). The control group (0 medications, *n* = 117, 25.66%) showed a median CET of 53 µm (IQR: 52–56 µm, range: 42–79 µm). Eyes receiving monotherapy (*n* = 147, 32.24%) had a median CET of 52 µm (IQR: 50–54 µm, range: 42–62 µm), dual therapy (*n* = 109, 23.90%) had a median CET of 50 µm (IQR: 48–53 µm, range: 32–68 µm), triple therapy (*n* = 57, 12.5%) had a median CET of 49 µm (IQR: 46–53 µm, range: 38–56 µm), and quadruple therapy (*n* = 26, 5.70%) had the lowest median CET of 48 µm (IQR: 45–49 µm, range: 22–57 µm).

### 2.3. CET by Specific Medication

**Figure 2:**
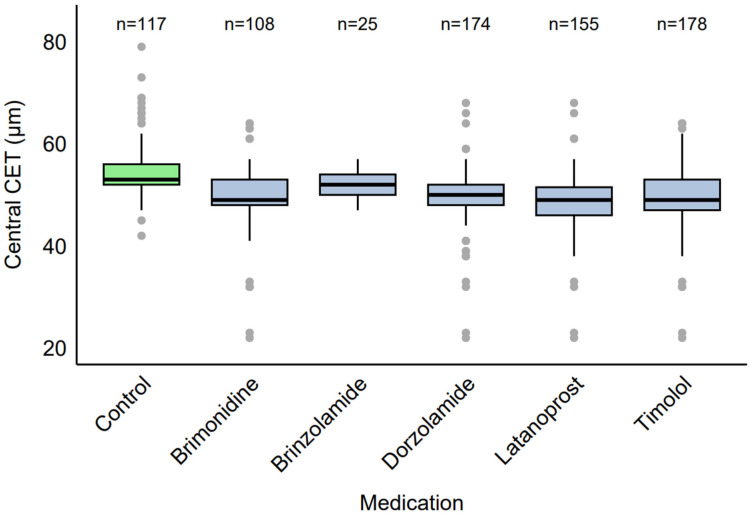
Distribution of CET by specific antiglaucoma medication.

Among the eyes receiving therapy (*n* = 339), common medication combinations were identified. When examined by specific medication (Figure 2), eyes using brimonidine (*n* = 108, 31.86%) had a median CET of 49 µm (IQR: 48–53 µm, range: 22–64 µm), brinzolamide (*n* = 25, 7.39%) had a median CET of 52 µm (IQR: 50–54 µm, range: 47–57 µm), dorzolamide (*n* = 174, 51.33%) had a median CET of 50 µm (IQR: 48–52 µm, range: 22–68 µm), latanoprost (*n* = 155, 45.72%) had a median CET of 49 µm (IQR: 46–51.5 µm, range: 22–68 µm), and timolol (*n* = 178, 52.51%) had a median CET of 49 µm (IQR: 47–53 µm, range: 22–64 µm).

### 2.4. Medication Combinations in Polytherapy

**Figure 3:**
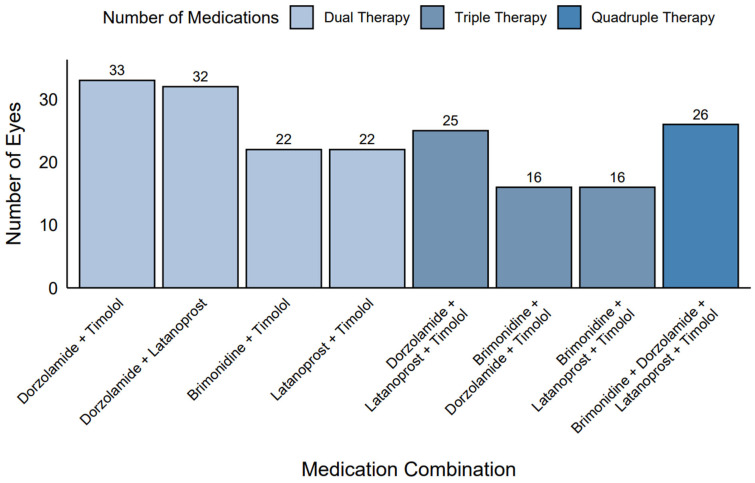
Frequency of medication combinations in polytherapy.

Among the eyes receiving polytherapy (*n* = 192), common medication combinations were identified (Figure 3). In the dual therapy group (*n* = 109), the most frequent combinations were dorzolamide + timolol (30.3%, *n* = 33), dorzolamide + latanoprost (29.4%, *n* = 32), brimonidine + timolol (20.2%, *n* = 22), and latanoprost + timolol (20.2%, *n* = 22). In the triple therapy group (*n* = 57), dorzolamide + latanoprost + timolol was the most common combination (43.9%, *n* = 25), followed by brimonidine + dorzolamide + timolol and brimonidine + latanoprost + timolol (28.1%, *n* = 16). All eyes in the quadruple therapy group (*n* = 26, 100%) used the combination of brimonidine + dorzolamide + latanoprost + timolol.

### 2.5. CET Correlation and Age Relationship

**Figure 4:**
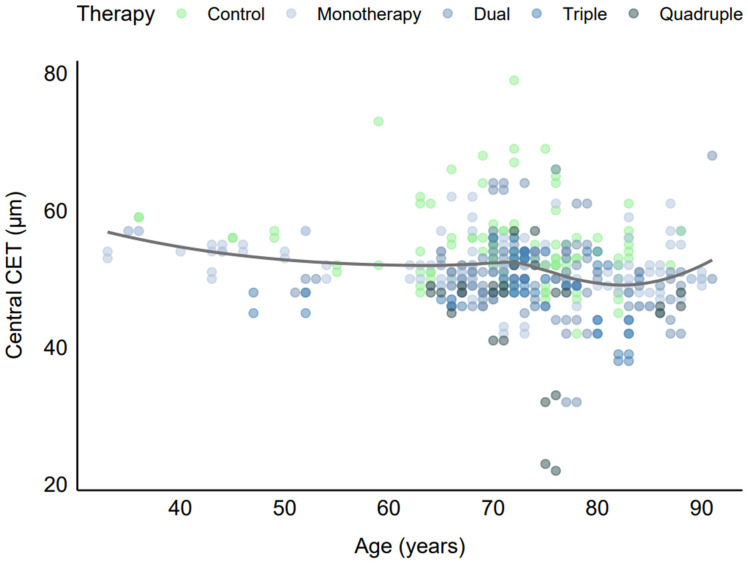
CET vs. age by number of antiglaucoma medications.

A strong inter-eye correlation in CET (Rho = 0.82) was observed, indicating high within-patient similarity. Spearman’s rank correlation coefficients showed weak associations between CET and age across medication groups: control (Rho = −0.17, *p* = 0.061, *n* = 117), monotherapy (Rho = −0.27, *p* = 0.001, *n* = 147), dual therapy (Rho = −0.06, *p* = 0.524, *n* = 109), triple therapy (Rho = −0.29, *p* = 0.027, *n* = 57), and quadruple therapy (Rho = −0.29, *p* = 0.145, *n* = 26). The monotherapy group’s significant correlation (*p* = 0.001) indicated a weak negative association, with older age linked to slightly thinner CET, though the effect size (Rho = −0.27) was modest. The triple therapy group also showed a significant weak negative association (*p* = 0.027, Rho = −0.29). In the quadruple therapy group, the correlation coefficient (Rho = −0.29) was identical to that of triple therapy, but the non-significant *p*-value (*p* = 0.145) was likely due to the smaller sample size (*n* = 26). Visual inspection of the scatter plot (Figure 4) revealed a relatively flat locally estimated scatterplot smoothing (LOESS) across the age range (33–90 years), confirming the absence of a consistent trend.

The lack of a strong age-related trend may be attributed to the dominant influence of antiglaucoma medications on CET, which likely overshadowed age-related corneal changes in this older cohort. Additionally, the limited representation of younger patients (minimum age 33 years) may have restricted the detection of age-related patterns. These findings indicate that age was not a major confounder in the relationship between medication use and CET, although the weak association in the monotherapy group warranted consideration in the multivariable regression analysis, which further adjusted for age and other covariates.

### 2.6. Multivariable Analysis of Antiglaucoma Medication Effects on Cet

The generalized estimating equation (GEE) model results presented in Table 1, based on 456 observations across 243 clusters, provide insights into the effects of antiglaucoma medications, age, and sex on CET, addressing objectives of evaluating CET differences, identifying medication impacts, and understanding the factors influencing corneal thickness. All five antiglaucoma medications were associated with statistically significant reductions in CET. Latanoprost had the largest effect, reducing CET by 3.01 µm (β = −3.01, 95% CI: −4.26 to −1.76, *p* < 0.001), followed by brinzolamide (β = −2.44, 95% CI: −4.35 to −0.52, *p* = 0.013) and brimonidine (β = −2.29, 95% CI: −3.80 to −0.78, *p* = 0.003). Dorzolamide and timolol showed smaller but significant reductions (β = −1.64, 95% CI: −2.81 to −0.47, *p* = 0.006; β = −1.67, 95% CI: −2.96 to −0.38, *p* = 0.012, respectively). Among all drugs, latanoprost caused the greatest CET reduction, highlighting its substantial epithelial impact. Age was a significant predictor, with each additional year (centered at the median) associated with a slight CET reduction of 0.07 µm (β = −0.07, 95% CI: −0.11 to −0.02, *p* = 0.002), consistent with the weak negative correlation in the monotherapy group (Rho = −0.27, *p* = 0.001), indicating a subtle age-related thinning effect of minimal clinical impact. Sex was not significant (β = 0.60, 95% CI: −0.76 to 1.95, *p* = 0.386), confirming no meaningful difference between males and females. The significant CET reductions by all medications highlight their potential to alter corneal epithelial properties, with latanoprost posing the greatest risk for thinning (β = −3.01 µm vs. dorzolamide’s β = −1.64 µm). Although these reductions appeared numerically modest (2–3 µm), they may reflect clinically relevant epithelial compromise over time. The model’s adjustment for the strong correlation between eyes (working correlation = 0.579) ensured robust estimates, accounting for within-patient clustering. The small age effect and lack of sex effect suggest that these are not major confounders, emphasizing medications as the primary drivers of CET changes.

**Figure 5:**
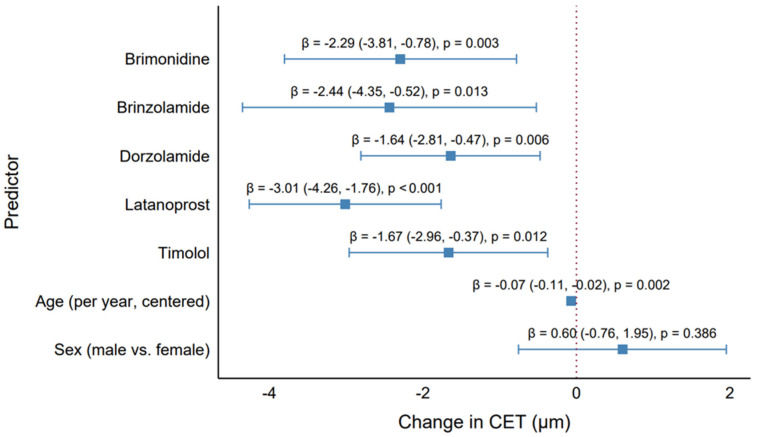
Effect of antiglaucoma medications on the central corneal epithelial thickness. Forest plot showing the GEE model estimates (β) and 95% confidence intervals for each medication (brimonidine, brinzolamide, dorzolamide, latanoprost, timolol), age (per year, centered), and sex (male vs. female), adjusted for all predictors.

The forest plot (Figure 5) enhanced interpretation by visually ranking the medications by effect size and significance, with labels above each point estimate providing precise β, CI, and *p*-value information. This visualization supports the study objective of identifying which medication had the greatest (latanoprost) and least (dorzolamide) impact on CET, guiding clinical considerations for medication selection in glaucoma management.

### 2.7. Multivariable Analysis of the Number of Antiglaucoma Medications on CET

**Figure 6:**
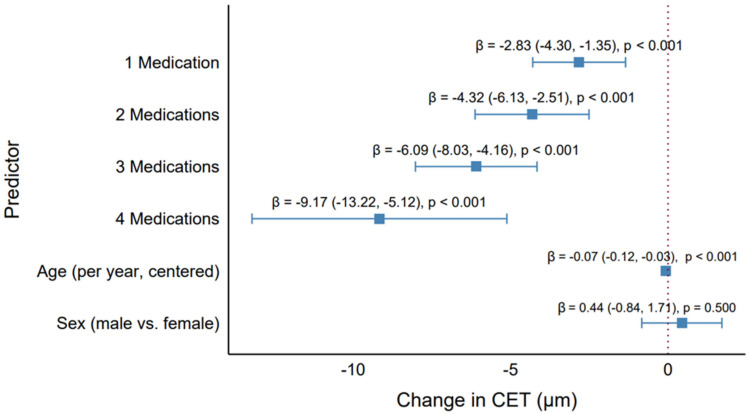
Effect of the number of antiglaucoma medications on the central corneal epithelial thickness. Forest plot showing the GEE model estimates (β) and 95% confidence intervals for the number of medications (1, 2, 3, 4 vs. control), age, and sex, adjusted for all predictors.

The GEE model assessed the effects of the number of antiglaucoma medications, age, and sex on CET, addressing the study objectives of evaluating the CET differences and investigating the impact of increasing medication numbers (Table 2, Figure 6). The results demonstrated a clear dose–response relationship, with a progressive reduction in CET as the number of medications increased, as visualized in the forest plot (Figure 6).

Compared with the control group (no medications), the use of one medication was associated with a significant CET reduction of 2.83 µm (95% CI: −4.30 to −1.35, *p* < 0.001). This effect intensified with additional medications: two medications reduced the CET by 4.32 µm (95% CI: −6.13 to −2.51, *p* < 0.001), three medications by 6.09 µm (95% CI: −8.03 to −4.16, *p* < 0.001), and four medications by 9.17 µm (95% CI: −13.22 to −5.12, *p* < 0.001). The forest plot illustrates this trend, with point estimates for the number of medications shifting progressively leftward (indicating greater CET reduction) and wider confidence intervals for higher medication counts, reflecting the smaller sample size for quadruple therapy (*n* = 26 eyes). The highly significant *p*-values (<0.001) across all medication levels underscore the robust association between polytherapy and CET thinning.

Age was also a significant predictor, with each additional year (centered at the median [75 years]) associated with a CET reduction of 0.07 µm (95% CI: −0.12 to −0.03, *p* < 0.001). This small but significant effect aligns with the weak negative correlation observed in the descriptive analyses (e.g., Rho = −0.27, *p* = 0.001 for monotherapy), revealing a subtle age-related thinning of the corneal epithelium. However, the clinical impact of this effect was minimal compared with the medication effects, as a 10-year age difference corresponded to only a 0.70 µm reduction in CET. Sex was not significant (β = 0.44 µm, 95% CI: −0.84 to 1.71, *p* = 0.500), with the forest plot showing its confidence interval crossing the null line (β = 0), indicating no meaningful difference between males and females.

The progressive CET reduction with increasing medication numbers revealed a cumulative effect of antiglaucoma medications. The largest effect observed with quadruple therapy (β = −9.17 µm) was clinically significant, as a 9 µm reduction was substantial relative to the median CET of 51 µm (IQR: 48–54 µm) in the cohort. This finding highlights the need for the careful monitoring of corneal health in patients on multiple medications, as significant thinning may impact the corneal barrier function or IOP measurements. The model’s adjustment for the strong correlation between eyes (Rho = 0.82) ensured robust estimates, accounting for within-patient clustering.

The forest plot (Figure 6) enhanced interpretation by visually depicting the dose–response relationship, with labels above each point estimate providing precise β, CI, and *p*-value information. These results demonstrate that the number of medications is a critical factor in CET changes, with implications for optimizing glaucoma treatment regimens to minimize corneal adverse effects.

## 3. Discussion

Numerous studies have demonstrated that antiglaucoma medications can significantly affect the structure and function of the corneal epithelium, with the nature of these changes varying depending on the class of drugs used [18,19,20]. However, there is a limited body of research examining epithelial alterations in the context of multi-drug therapy—particularly dual and triple therapy. There is limited evidence regarding whether the number of concurrently administered medications correlates with the extent of structural alterations in the corneal epithelium. A thorough analysis of this issue is essential to advance the understanding of the impact of multi-drug therapy on ocular surface health.

This study aimed not only to quantify the epithelial changes associated with topical antiglaucoma therapy, but also to identify patient subgroups particularly vulnerable to such effects. In light of the observed dose-dependent thinning of the corneal epithelium, special attention should be given to individuals receiving two or more medications simultaneously, as they may be at increased risk of ocular surface compromise. By analyzing both the number and type of administered drugs, our findings offer new insights into the mechanisms underlying structural alterations in the epithelium and highlight potential risk factors associated with prolonged multi-drug therapy.

In this cross-sectional cohort of 242 adults (456 eyes; 58% female; median age 72 years, IQR 68–78), the central corneal epithelial thickness exhibited a clear dose-response, falling from 53 µm in the untreated controls to 52, 50, 49, and 48 µm with mono-, dual-, triple-, and quadruple-topical therapy, respectively. Generalized estimating equations confirmed a cumulative reduction of 9 µm under quadruple treatment (*p* < 0.001). Latanoprost exerted the most pronounced single- drug effect (−3.0 µm), whereas β-blockers and carbonic anhydrase inhibitors produced only modest thinning (≈1–2 µm). Age and sex did not meaningfully influence the thickness. These findings are consistent with previous studies that documented significant epithelial thinning after chronic antiglaucoma therapy [9,18,21,22,23,24].

Although the absolute changes in the central corneal epithelial thickness (CET) observed across treatment groups—ranging from 53 µm in the controls to 48 µm in patients on quadruple therapy—may appear numerically small (1–2 µm between adjacent groups), they are not necessarily clinically trivial. The OCT-derived epithelial thickness is a highly reproducible and sensitive marker of ocular surface integrity. Several studies suggest that even a 1–2 µm reduction may reflect early epithelial stress or compromise, particularly when sustained over time and occurring in a cumulative, dose-dependent pattern. Notably, CET thinning correlates with the signs and symptoms of ocular surface disease (OSD) including tear film instability, epithelial fragility, and reduced patient comfort—factors that can significantly impair quality of life and adherence to glaucoma therapy. While no universal threshold exists for clinically meaningful CET decline, longitudinal studies have linked reductions as small as 2–3 µm to increased OSDI scores and fluorescein staining. Despite being subtle, the observed changes in CET may serve as potential biomarkers of subclinical toxicity. Regular CET monitoring could therefore aid in the early identification of patients at risk for ocular surface complications, supporting timely interventions such as switching to preservative-free formulations or surgical options to preserve epithelial health.

Due to the composition of our study cohort, we only included patients treated with preserved antiglaucoma medications, primarily containing benzalkonium chloride (BAK). Preservative-free therapies were excluded due to their minimal representation in multi-drug regimens, which would have precluded a reliable analysis of the relationship between the number of medications and CET. This strategy minimized the influence of uncontrolled variables and allowed for a clearer assessment of the pharmacological effects attributable to active substances. This decision was also consistent with the study’s aim, which was to evaluate the toxicity of active antiglaucoma agents, rather than to compare the effects of formulations with and without preservatives.

Although we did not directly analyze the type or concentration of preservatives in individual formulations, benzalkonium chloride (BAK) remains the most widely used and well-studied compound in topical antiglaucoma medications. Its proinflammatory and cytotoxic properties—mediated through the disruption of epithelial junctions, increased oxidative stress, and apoptosis—have been repeatedly demonstrated in both in vitro and in vivo models. In particular, BAK has been shown to impair limbal stem cell function [25], disrupt tear film stability and epithelial integrity [26], and induce cell death via inflammatory and oxidative stress-related pathways [27]. Therefore, the observed epithelial thinning may be attributable not only to the pharmacological activity of the medications, but also to the cumulative epithelial toxicity of BAK.

Importantly, different topical antiglaucoma medications may contain not only various concentrations of BAK, but also different chemical forms or preservative systems, which could influence their toxic potential. This heterogeneity may contribute to the differences in epithelial impact observed between drug classes and individual agents. Future research should therefore address not only the presence or absence of preservatives, but also their quantitative and qualitative characteristics—particularly in relation to long-term corneal epithelial health. Prospective studies comparing preserved and preservative-free formulations in real-world multi-drug regimens would be instrumental in clarifying the contribution of preservative-related toxicity to the chronic ocular surface damage seen in glaucoma patients.

In the study by Nam et al. [19], the average difference in CET between glaucomatous and healthy eyes was 4.2 µm. The authors emphasized the significance of the number of medications and the presence of BAK, which supports our findings indicating a strong association between medication count and CET thinning. Similarly, Batawi et al. [8] reported a negative correlation between the number of medications and CET. In contrast, Halkiadakis et al. [20] found CET thinning in treated patients but did not establish a clear relationship with the treatment parameters, possibly due to methodological differences or a smaller sample size.

Analyzing the effects of specific drugs, our results indicated that latanoprost had the most pronounced impact. This is consistent with numerous experimental and clinical studies [19,28,29,30,31]. Both in vitro and in vivo models have demonstrated that latanoprost—especially in BAK-containing formulations—induces increased epithelial apoptosis and disrupts tight junction proteins (ZO-1, occludin), thereby compromising epithelial barrier function [32,33]. Additionally, an increased activity of matrix metalloproteinases (MMP-3, -9), which degrade corneal structure, has been observed with 0.005% preservative [33]. Importantly, some of these effects, such as a reduced expression of tight junction proteins and enhanced apoptosis, were also noted with preservative-free formulations, suggesting that the epithelial impact may also stem from the active compound itself [33]. In vivo studies have further confirmed that chronic exposure to BAK-containing latanoprost reduces the superficial epithelial cell density and remodels the basal epithelial layer [34].

It is worth emphasizing that although β-blockers and carbonic anhydrase inhibitors also affected the CET, the extent was significantly smaller. In our model, dorzolamide and brinzolamide led to less pronounced, albeit statistically significant, thinning. Nam et al. [19] and Martone et al. [34] suggest that the presence of BAK, rather than the active ingredient itself, may be the primary toxic factor in some drugs—particularly β-blockers. This view was reinforced by a randomized trial by Kim et al., in which switching from a BAK-preserved to preservative-free brimonidine/timolol fixed combination significantly improved the OSDI scores and corneal staining while maintaining an identical IOP-lowering efficacy [35]. Similarly, a large epidemiological study by Pisella et al. (>4000 glaucoma patients) reported more than twice the incidence of ocular-surface symptoms with BAK-containing drops and rapid symptom relief after conversion to preservative-free formulations [36].

Clinical data suggest that fixed combinations containing brimonidine may offer superior ocular comfort compared with dorzolamide-based regimens, which may enhance long-term compliance in glaucoma patients [37,38]. However, despite its favorable tolerability profile, in vitro studies have indicated that brimonidine may exert cytotoxic effects on corneal epithelial cells. Kucukoduk et al. demonstrated that preservative-free brimonidine induced significantly stronger early apoptosis than preserved formulations [39], and Fisenko et al. reported that high-concentration brimonidine (1:10 dilution), even without BAK, caused morphological damage and reduced cell viability [40]. Similarly, timolol at comparable dilutions exhibited cytotoxic effects only when preserved with BAK, while preservative-free timolol showed minimal toxicity. This corresponds with our findings, where timolol use led to a small but significant CET reduction, likely reflecting the cumulative impact of chronic exposure [40].

Carbonic anhydrase inhibitors (CAIs), such as dorzolamide and brinzolamide, generally exert weaker epithelial effects compared with prostaglandin analogues. However, in vitro data indicate they can still reduce epithelial cell proliferation and induce apoptosis, as shown by Pozarowska et al. in a dose-dependent manner [41]. Fisenko et al. also observed that unpreserved dorzolamide, at higher dilutions (1:10–1:50), led to decreased epithelial cell viability and morphological alterations [40]. Importantly, most of the cytotoxicity appears to be related to preservatives rather than the active compounds themselves.

These findings underscore that while β-blockers, CAIs, and brimonidine are often considered better tolerated than prostaglandin analogues, they can still contribute to epithelial compromise—especially when used chronically or in multi-drug regimens. Monitoring corneal epithelial thickness may therefore be useful in long-term glaucoma management.

Ocular and systemic comorbidities may also impact epithelial measurements and contribute to interindividual variability. For example, dry eye disease has been associated with peripheral epithelial irregularity and epithelial damage, particularly in more severe forms [42], while type 2 diabetes mellitus has been linked to significantly reduced central epithelial thickness due to impaired regeneration and chronic metabolic stress [43]. Although patients with these conditions were excluded from our study to minimize confounding, they may represent vulnerable subgroups in clinical practice. Future studies should evaluate these populations more specifically.

The relationship between the number of medications and CET in our study was clear and linear. Each additional substance resulted in further statistically significant epithelial thinning, corroborating findings from other authors. Ye et al. [9] demonstrated that CET decreases as the number of topical antiglaucoma medications increases, particularly in patients receiving three or more agents. Similar findings were reported by Batawi [8] and also by Zahoor et al., who showed in a prospective study that patients using a greater number of medications (up to four) and more frequent daily dosing (up to six times) experienced more pronounced epithelial thinning within one year of treatment [44].

These findings suggest that the additive effect is not solely due to the pharmacodynamics of individual agents, but may also reflect cumulative toxicity from repeated daily exposure of the epithelium to multiple drug classes.

Ultimately, the findings of this research are expected to not only expand knowledge about the effects of antiglaucoma therapy on the ocular surface, but also to support the development of more effective strategies for managing glaucoma treatment and preventing corneal complications.

Several limitations of this study should be acknowledged for the accurate interpretation of results. First, the cross-sectional nature of our study inherently limits causal inference. While we demonstrated a statistically significant association between antiglaucoma medication use and reduced CET, we cannot exclude the possibility that some degree of epithelial alteration existed prior to treatment initiation. Definitive conclusions regarding causality would require prospective longitudinal studies capturing baseline CET measurements and subsequent changes over time. Thus, caution is warranted in interpreting these findings and highlight the need for future longitudinal research to validate and expand upon these observations.

Moreover, the imaging modality used—anterior segment optical coherence tomography (AS-OCT)—despite being non-invasive and reproducible, has resolution limitations. Only the total epithelial thickness was measured, without distinguishing between the superficial and basal layers. Confocal studies (e.g., Martone et al.) have shown that changes may primarily affect the superficial layer, while the basal layer may become denser [34]. Our measurements averaged these changes—OCT maps thickness but does not provide cellular-level detail. In the study by Cennamo et al. (2018) [18], advanced corneal epithelial damage assessed using scanning electron microscopy (SEM) revealed ultrastructural changes that were not always reflected in the OCT-derived CET. The authors observed that even with significant microvilli alterations, the CET may remain unchanged, highlighting the limitations of OCT in detecting specific epithelial injuries [18].

Another limitation is that patients with relevant ocular and systemic comorbidities, such as dry eye, corneal dystrophies, and diabetes, were excluded from the study. While this helped reduce confounding, it limited the ability to assess whether certain subgroups are more susceptible to epithelial changes. Future studies should aim to investigate these populations more directly.

Despite these limitations, the findings have important clinical implications. First, they underscore that ocular surface toxicity is a relevant concern in chronically treated glaucoma patients. CET thinning may serve as a marker of chronic ocular surface damage caused by topical therapy. Although differences of a few microns in the CET may not substantially affect the IOP measurement (unlike central corneal thickness, CCT), they reflect a broader phenomenon—drug-induced dry eye disease. A compromised and thinned epithelium translates into reduced tear film stability and can manifest as irritation symptoms such as burning, foreign body sensation, and redness. Nearly half of glaucoma patients on drops experience ocular surface disease symptoms, which negatively affect their quality of life and adherence to treatment [45].

Our results offer an objective parameter (CET measured by OCT) to monitor these changes, which may be helpful for inpatient follow-up. Regular corneal assessment (e.g., periodic CET measurements) in glaucoma patients may help detect early epithelial abnormalities and prompt timely intervention before complications arise. Severe epithelial damage can lead to punctate erosions and recurrent corneal defects, increasing the risk of infection or ulceration. Moreover, ocular discomfort and pain may reduce treatment adherence—paradoxically, vision-saving drops may be discontinued due to poor tolerance. In such cases, therapy modification should be considered—reducing the number of medications (if target IOP can be maintained), switching to better-tolerated compounds, and implementing ocular surface support (e.g., adding lubricants or nighttime protection).

Such approaches align with the current recommendations for managing adverse effects in chronic glaucoma therapy—as highlighted by Inoue [46], reducing the number of drops, decreasing the dosing frequency, and choosing more tolerable formulations can significantly improve patient comfort and treatment compliance. This reflects a modern, holistic approach to glaucoma care, where ocular surface preservation and patient quality of life are as important as IOP control.

## 4. Materials and Methods

### 4.1. Study Design and Population

This cross-sectional study included 456 eyes from 242 patients, evaluated between February 2024 and January 2025 at the Department of Ophthalmology, Saint Barbara Hospital, Trauma Center, Sosnowiec, Poland. All study procedures adhered to the tenets of the Declaration of Helsinki and were approved by the Institutional Ethics Committee (Approval No. 27/KB/AŚ/04/2024). Written informed consent was obtained from all participants prior to enrollment.

Patients were allocated into five groups according to the number of topical antiglaucoma medications used: a control group (no treatment), and treatment groups receiving one (monotherapy), two (dual therapy), three (triple therapy), or four (quadruple therapy) antiglaucoma agents. Each treated patient had received their current regimen continuously for at least six months prior to central corneal epithelial thickness (CET) assessment. The control group consisted of 59 healthy individuals (117 eyes) with no history of antiglaucoma therapy, while 183 individuals (339 eyes) formed the pharmacologically treated cohort.

Only patients using topical antiglaucoma medications were eligible for inclusion. The use of any additional topical ocular agents, including artificial tears, corticosteroids, antibiotic drops, or ophthalmic ointments, was an exclusion criterion. Medication history was verified based on current physician-issued treatment instructions, electronic medical records, and direct patient interview. All patients were under regular ophthalmologic care at our institution, which allowed for the consistent and reliable assessment of treatment status and ensured that no co-medications potentially affecting the ocular surface were present at the time of inclusion. Patients were eligible for inclusion only if their IOP was within the normal physiological range of 10–21 mmHg, regardless of the treatment group.

A detailed breakdown of the eyes by treatment group and medication combination is presented below:

- Monotherapy (147 eyes): dorzolamide (42), brimonidine (28), latanoprost (34), timolol (18), and brinzolamide (25).
- Dual therapy (109 eyes): brimonidine + timolol (22), dorzolamide + latanoprost (32), dorzolamide + timolol (33), and latanoprost + timolol (22).
- Triple therapy (57 eyes): brimonidine + dorzolamide + timolol (16), brimonidine + latanoprost + timolol (16), and dorzolamide + latanoprost + timolol (25).
- Quadruple therapy (26 eyes): brimonidine + dorzolamide + latanoprost + timolol.

Each eye was independently assigned to a treatment group based on the type and number of medications administered at the time of evaluation. GEEs were employed to account for within-patient inter-eye correlation during the statistical analyses.

Patients who had undergone any ocular surgery within the past two years were excluded from the study. For the purposes of this analysis, ocular surgery was defined broadly to include both incisional and laser-based procedures. Specifically, we excluded patients with a history of laser trabeculoplasty (ALT or SLT), peripheral laser iridotomy, micropulse transscleral cyclophotocoagulation (MP-TSCPC), continuous-wave transscleral cyclophotocoagulation (TSCPC), and minimally invasive glaucoma surgeries (MIGSs) such as iStent or Hydrus implantation. The only exceptions permitted were uncomplicated prior cataract surgery and posterior capsulotomy performed with an Nd:YAG laser, provided that a minimum interval of two years had elapsed since the procedure.

Although exact data on pseudophakia were not collected, the two-year surgery-free requirement was intended to minimize the impact of prior cataract surgery on epithelial thickness. Furthermore, while the overall duration of glaucoma diagnosis was not documented, all treated patients had been using their current topical medication regimen for at least six months, ensuring that any epithelial changes observed reflected chronic exposure.

Control group subjects were recruited from individuals undergoing routine ophthalmological examinations who had no history of glaucoma, ocular hypertension, or other chronic ocular surface diseases. All participants had normal intraocular pressure and no history of any ocular surgical procedures other than uncomplicated cataract surgery; however, individuals who had undergone cataract extraction within the previous two years were excluded. None of the individuals in the control group used any topical ophthalmic medications, including artificial tears, for at least six months prior to examination. Comprehensive slit-lamp examination confirmed the absence of corneal epithelial irregularities or signs of ocular surface disease (e.g., conjunctival hyperemia, punctate keratitis).

The inclusion and exclusion criteria applied to ensure homogeneity and eliminate potential confounders are summarized in Table 3.

### 4.2. Ophthalmic Examination and Imaging Protocol

All subjects underwent comprehensive ophthalmic examination. Best-corrected distance visual acuity (BCVA) was evaluated using a standard Snellen chart. IOP was measured using air puff non-contact tonometry. Goldmann applanation tonometry was intentionally avoided to prevent potential disturbance of the corneal surface and maintain the integrity of epithelial thickness measurements. All IOP assessments were performed with the same calibrated device following a standardized protocol. Anterior and posterior segment evaluations were performed using slit-lamp biomicroscopy (BQ 900, Haag-Streit AG, Köniz, Switzerland) and dilated fundus examination (with 1% tropicamide; % Tropicamide WZF, Polpharma, Warsaw, Poland), respectively. Importantly, optical coherence tomography (OCT; Optovue, Fremont, CA, USA) imaging was conducted prior to mydriasis to avoid potential interference with the epithelial thickness measurements.

### 4.3. Measurement of Corneal Epithelial Thickness

The central corneal epithelial thickness was assessed using spectral-domain optical coherence tomography (SD-OCT) with the SOLIX platform (Optovue, Fremont, CA, USA). All measurements were performed by a single experienced operator to minimize inter-examiner variability. The device’s 10 mm corneal layer mapping protocol enabled high-resolution imaging and quantitative assessment of the epithelial, stromal, and total corneal thickness across 16 radial meridians.

Advanced imaging tools such as the Highlight feature facilitated the detection of subtle epithelial irregularities, while the integrated Change Analysis module supported longitudinal comparison. The embedded normative database allowed for population-based referencing to enhance diagnostic reliability and precision.

### 4.4. Statistical Analysis

Statistical analyses were performed using R software (version 4.3.3). Descriptive statistics included medians, interquartile ranges, and frequency distributions. GEEs with exchangeable correlation structure were applied to account for within-subject correlations between eyes. Two separate GEE models were constructed: the first assessed the independent effects of five antiglaucoma medications, and the second examined the impact of the number of medications (0 to 4) on the CET. Age and sex were included as covariates in both models. The normality assumption of the CET was checked via histograms and Q–Q plots, while the intraclass correlation coefficient (ICC = 0.82) justified the model structure. Spearman’s correlation and VIF were used to assess the collinearity and age effects. Results were reported with β coefficients, 95% confidence intervals, and *p*-values, with *p* < 0.05 considered statistically significant.

## 5. Conclusions

Taken together, our findings indicate that cumulative topical antiglaucoma therapy—rather than patient age or sex—is the primary determinant of corneal epithelial thinning, with latanoprost exerting the most pronounced single-drug effect. In patients with coexisting moderate to severe dry eye disease, early de-escalation to preservative-free fixed combinations or minimally invasive glaucoma surgery (MIGS) should be considered. Importantly, ET could serve as an objective biomarker to guide treatment adjustments and preserve ocular surface integrity [48]. Supporting this approach, a recent two-year phase I/II study demonstrated that a single intracameral bimatoprost sustained-release implant could maintain the target IOP while offering superior ocular surface preservation compared with daily topical therapy [49]. Prospective multicenter trials are now warranted to determine whether CET-guided treatment algorithms improve the long-term patient comfort, adherence, and visual outcomes.

Further research should evaluate whether the adjunctive use of ocular lubricants mitigates epithelial thinning and improves tolerability in patients on chronic multi-drug regimens. In addition, the individual and synergistic effects of different antiglaucoma drug classes on epithelial morphology and ocular surface integrity warrant detailed investigation. Future studies should also account for the duration and severity of glaucoma, as more advanced disease and prolonged treatment exposure may be associated with more pronounced epithelial alterations.

## Figures and Tables

**Table 1:** GEE model results for CET, adjusted for age and sex.

Predictor	Estimate, β (µm)	SE	95% CI	*p*
Intercept (control, female patient at age 72 years)	54.09	0.53	53.04, 55.14	<0.001
Brimonidine (yes vs. no)	−2.29	0.77	−3.80, −0.78	0.003
Brinzolamide (yes vs. no)	−2.43	0.98	−4.35, −0.52	0.013
Dorzolamide (yes vs. no)	−1.64	0.60	−2.80, −0.47	0.006
Latanoprost (yes vs. no)	−3.01	0.64	−4.26, −1.76	<0.001
Timolol (yes vs. no)	−1.67	0.66	−2.96, −0.37	0.012
Age (per year, centered)	−0.07	0.02	−0.11, −0.02	0.002
Sex (male vs. female)	0.60	0.69	−0.76, 1.95	0.386

**Table 2:** GEE model results for CET, adjusted for number of medications, age, and sex.

Predictor	Estimate (µm)	SE	95% CI	*p*-Value
Intercept (control, female patient at age 72 years)	54.40	0.63	53.16; 55.63	<0.001
One medication (vs. control)	−2.83	0.75	−4.30, −1.35	<0.001
Two medications (vs. control)	−4.32	0.92	−6.13, −2.51	<0.001
Three medications (vs. control)	−6.09	0.99	−8.03, −4.16	<0.001
Four medications (vs. control)	−9.17	2.07	−13.22, −5.12	<0.001
Age (per year, centered)	−0.07	0.02	−0.12, −0.03	<0.001
Sex (male vs. female)	0.44	0.65	−0.84, 1.71	0.500

**Table 3:** Inclusion and exclusion criteria.

Inclusion Criteria	Exclusion Criteria
Age > 18 years	History of ocular surface disease (e.g., dry eye syndrome)
Use of 0–4 topical antiglaucoma medications continuously for ≥6 months	Presence of corneal pathology (e.g., dystrophy, degeneration, scarring, or neovascularization)
Ability to provide written informed consent	History of ocular trauma or uveitis
	Systemic disease with ocular manifestations (e.g., diabetes mellitus, connective tissue disorders)
	History of any ocular surgery, including cataract extraction or glaucoma surgery, within the past two years [47]
	Concurrent use of artificial tears or ocular lubricants

## Data Availability

The data presented in this study are available on request from the corresponding author. The data are not publicly available due to third-party rights and commercial confidentiality.
